# Parliament and Publications

**Published:** 1911-01-07

**Authors:** 


### Parliament and Publications.

### Report of the Medical Officer to the Local Government Board.

This report is chiefly a record of the work carried out under the general direction of the Board's medical officer during the year ended March 31, 1910, and relates to the conditions affecting the public health in England and Wales, with which the Board are concerned as the Central Public Health Authority. Much of the matter contained in this volume has been published previously in the form of separate reports, which have already been dealt with in The Hospital.—"Thirty-ninth annual report of the Local Government Board, supplement containing the report of the medical officer," Cd. 5312, price 3s. 7d.

